# Deep learning-based segmentation of the lung in MR-images acquired by a stack-of-spirals trajectory at ultra-short echo-times

**DOI:** 10.1186/s12880-021-00608-1

**Published:** 2021-05-08

**Authors:** Andreas M. Weng, Julius F. Heidenreich, Corona Metz, Simon Veldhoen, Thorsten A. Bley, Tobias Wech

**Affiliations:** Department of Diagnostic and Interventional Radiology, University Hospital Würzburg, Oberdürrbacher Str. 6, 97080 Würzburg, Germany

**Keywords:** MRI, Lung, Deep learning, Image segmentation

## Abstract

**Background:**

Functional lung MRI techniques are usually associated with time-consuming post-processing, where manual lung segmentation represents the most cumbersome part. The aim of this study was to investigate whether deep learning-based segmentation of lung images which were scanned by a fast UTE sequence exploiting the stack-of-spirals trajectory can provide sufficiently good accuracy for the calculation of functional parameters.

**Methods:**

In this study, lung images were acquired in 20 patients suffering from cystic fibrosis (CF) and 33 healthy volunteers, by a fast UTE sequence with a stack-of-spirals trajectory and a minimum echo-time of 0.05 ms. A convolutional neural network was then trained for semantic lung segmentation using 17,713 2D coronal slices, each paired with a label obtained from manual segmentation. Subsequently, the network was applied to 4920 independent 2D test images and results were compared to a manual segmentation using the Sørensen–Dice similarity coefficient (DSC) and the Hausdorff distance (HD). Obtained lung volumes and fractional ventilation values calculated from both segmentations were compared using Pearson’s correlation coefficient and Bland Altman analysis.

To investigate generalizability to patients outside the CF collective, in particular to those exhibiting larger consolidations inside the lung, the network was additionally applied to UTE images from four patients with pneumonia and one with lung cancer.

**Results:**

The overall DSC for lung tissue was 0.967 ± 0.076 (mean ± standard deviation) and HD was 4.1 ± 4.4 mm. Lung volumes derived from manual and deep learning based segmentations as well as values for fractional ventilation exhibited a high overall correlation (Pearson’s correlation coefficent = 0.99 and 1.00). For the additional cohort with unseen pathologies / consolidations, mean DSC was 0.930 ± 0.083, HD = 12.9 ± 16.2 mm and the mean difference in lung volume was 0.032 ± 0.048 L.

**Conclusions:**

Deep learning-based image segmentation in stack-of-spirals based lung MRI allows for accurate estimation of lung volumes and fractional ventilation values and promises to replace the time-consuming step of manual image segmentation in the future.

## Background

Four of the top ten global causes of deaths in 2016 were related to lung diseases: chronic obstructive pulmonary disease (COPD), lower respiratory tract infections, cancer and tuberculosis [1]. Imaging of the lungs thereby represents an important diagnostic tool for initial diagnosis and disease management. To date, the gold standard methodologies for lung imaging are computed tomography (CT) and conventional radiography; however, due to constant developments, magnetic resonance (MR) imaging evolves as a promising alternative for radiation-free imaging of the lungs [2].

In the last years, several studies investigated the feasibility of MR imaging for assessment of functional parameters, i.e. ventilation and/or perfusion [3–8]. Contrast-enhanced approaches based on gadolinium chelate complexes [8, 9], noble [10, 11] or fluorinated gases [12] have been proposed, while Fourier decomposition [3, 13] and Self-gated Non-Contrast-Enhanced Functional Lung imaging (SENCEFUL) [4, 14] represent methods which completely waive the administration of any contrast-agent. Recently, SENCEFUL was combined with ultra-short echo time (UTE) imaging [15] to yield higher signal gain from lung tissue compared to standard non-UTE imaging sequences [16]. Furthermore, a stack-of-spirals trajectory [17], as introduced and thoroughly compared to the spherical counterpart for lung imaging in Dournes et al. [18], has been applied to significantly shorten the overall scan duration for UTE-based functional lung MRI [19, 20]. Besides improved scan-times, however, post-processing for functional image analysis is cumbersome up to now. In particular, lung segmentation is required for an overall quantification of functional lung parameters like fractional ventilation and the determination of lung volumes for different breathing states. Due to varying signal intensities, image artifacts and non-isotropic image resolution, automatic approaches based on thresholding or region-growing are prone to errors, such that tedious and time-consuming manual segmentation has most commonly been preferred in the past. In recent years, semi-automatic approaches [4, 13, 15] were proposed to reduce the user interaction and efforts have also been made to fully automate the segmentation step by constructing and applying a library of manually segmented lung atlases [21].

With the growing success of exploiting machine learning in general, a plethora of post-processing techniques based on artificial neural networks (ANN) has also been proposed for medical imaging lately. Since, empirically, the human eye seems to be able to discriminate the lung parenchyma from other tissues regardless of inhomogeneities or image artifacts quite well, and ANNs are particularly well suited for perceptual tasks, corresponding methods have lately been implemented and tested also for lung imaging with promising results [22–27].

In this study, a 2D convolutional neural network (CNN) was trained and tested for semantic segmentation of lung images obtained from stack-of-spirals based UTE examinations to significantly shorten and simplify the post-processing workload for the fast functional lung MR imaging technique proposed in [20].

## Methods

The study was approved by the local ethics committee and written informed consent was obtained from every participant prior to inclusion.

### MR imaging and manual image segmentation

An image database was assembled from mid-2018 until mid-2019, comprising a total of 25,047 two-dimensional MR images of the lung in coronal orientation. The database originates from 53 examinations (33 healthy volunteers, 20 patients suffering from cystic fibrosis (CF)), each comprising a 3D coverage of the lung in 5 different breathing depths from deep expiration to deep inspiration. Each of these individual respiratory phases was acquired in a respective breath-hold of the participant. This approach is typically performed to determine lung volumes as well as fractional ventilation values as suggested in [20, 28].

All examinations were performed on a clinical 3 T MRI scanner (MAGNETOM Prisma, Siemens Healthcare, Germany) using a 3D UTE sequence based on a stack-of-spirals trajectory [17]. The latter applies spiral read-outs in two dimensions (coronal orientation in our study) and phase encoding in the remaining one. UTE contrast is enabled by minimizing the length of each individual phase encoding gradient, leading to shortest echo-times TE_min_ in the center of k-space and increasing echo-times towards higher partitions / values of k. This trajectory is a promising faster alternative to koosh-ball-like approaches [15], as the latter exhibit extreme and therefore time-consuming oversampling in the center of k-space. Furthermore, stack-of-spirals allow an anisotropic FOV, such that the dorsal–ventral dimension of the thorax can be scanned with a smaller FOV than the remaining dimensions. The following imaging parameters were used: TE_min_ = 0.05 ms; TR = 2.35 ms; flip angle = 5°; in-plane resolution = 2.3 × 2.3 mm; slice thickness = 2.3 mm; number of spiral readouts per partition = 264. The number of acquired partitions depended on the individual thorax size of the subject. In order to scan the whole volume in one breath hold, 6/8 partial-Fourier imaging was used in slice encoding direction resulting in a scan time of ~ 14 s per breathing state. SPIRiT [29] was applied for image reconstruction (acceleration factor = 2).

**Fig. 1:**
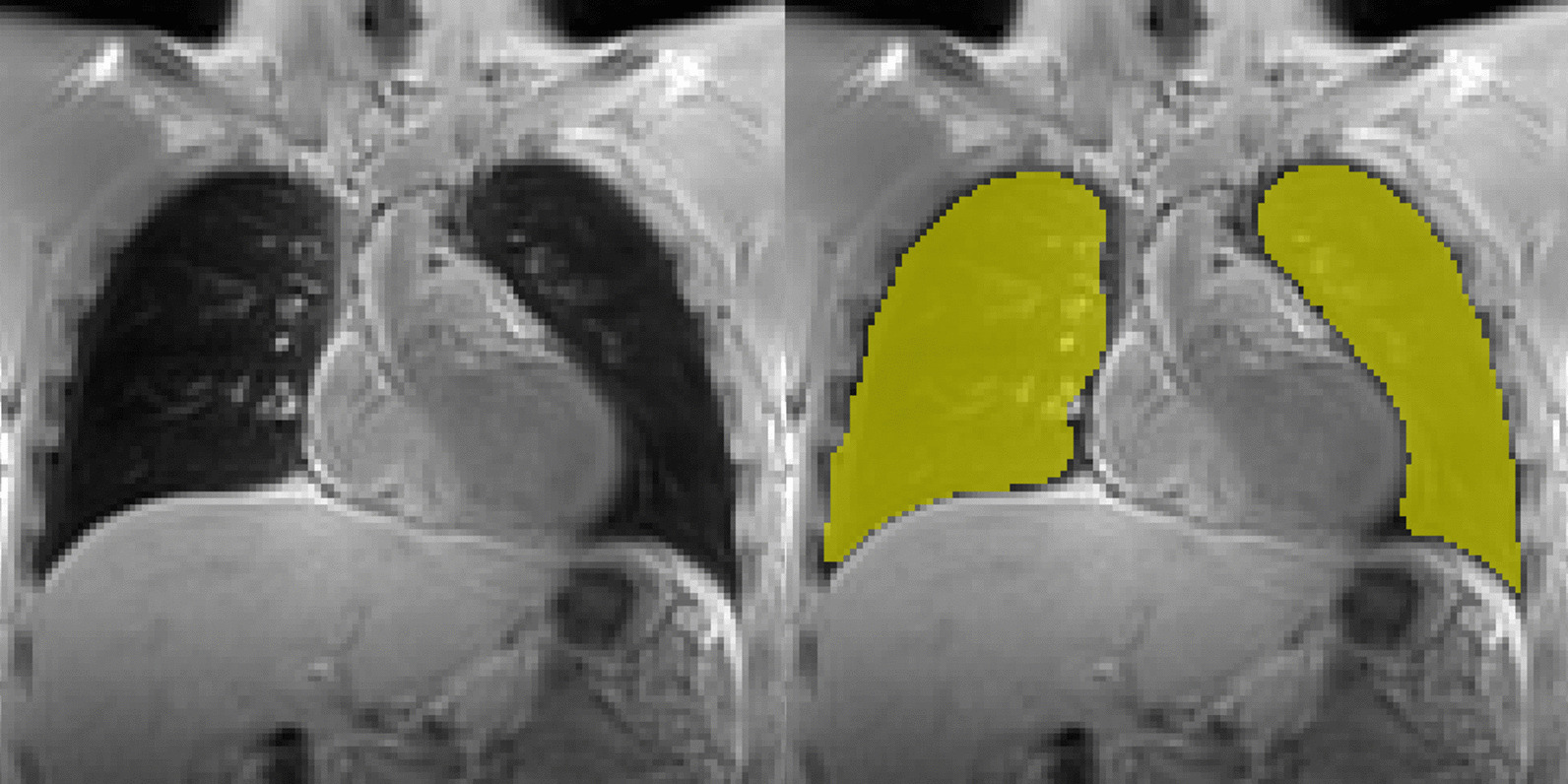
Representative morphologic image (left) with superimposed labels of manual segmentation (right)

The obtained images were segmented manually by an experienced user (radiologist with 2 + years of experience in lung MRI) via an in house-built segmentation tool allowing for manual delineation of the lung (ground truth). In the resulting binary 2D masks each pixel was assigned either to class ‘lung’ or ‘background’. An example of a 2D image and the corresponding manual segmentation is presented in Fig. 1.

### Convolutional neural network for semantic segmentation

A 2D convolutional neural network (CNN) was developed and trained to automatically perform semantic segmentation of UTE lung images. A SegNet architecture [30] was implemented in MATLAB (Ver 2019b, Deep Learning Toolbox, The MathWorks, Natick, MA, USA), and weights were initialized by those from the VGG-16 network. Exploiting weights from a network trained for image-handling—even though, not explicitly for the special case targeted here—has been reported to result in faster convergence of the training than a random initialization [31].

The CNN was trained using a subset of 17,713 2D images in coronal orientation and corresponding labels from manual segmentation. An additional validation dataset consisting of 2414 images was used for an unbiased evaluation during training, predominantly to avoid overfitting. Adaptive moment estimation (ADAM) was used as training optimizer and cross entropy as loss function. The initial learning rate was set to 5e−4, with a scheduled learning rate drop each 20 epochs with a drop factor of 0.95. Class weights were applied to address the imbalance of the classes. The CNN was trained for 588 epochs until the Sørensen-Dice similarity coefficient (DSC) and cross entropy loss of the validation set reached a steady state and validation did not yet indicate overfitting. The final model was then used for segmentation of the test subset. Training and evaluation of the network was performed on a personal computer (Intel Core i7-3820 CPU @ 3.6 GHz, 64 GB RAM and a NVIDIA Titan XP GPU).

### Evaluation of segmentation results

The performance of the trained 2D-CNN was evaluated by use of 4920 independent 2D test images, which were neither part of the training nor the validation data. Test data contained examinations from 5 healthy controls and 5 CF-patients each consisting of 5 different breathing states.

### Statistical analysis

Global accuracy of the network was assessed by dividing the absolute number of correctly classified pixels by the number of all pixels in the dataset. For the two labels lung and background, class accuracy was calculated as the ratio of correctly classified pixels to the total number of pixels in that class, according to the ground truth. The performance of the network in terms of lung detection in general was assessed via calculation of the number of true positive cases (TP, lung tissue detected in both manual and automatic approach), true negative cases (TN, no lung label in neither of the two approaches), false positive cases (FP, lung detected by the network while no lung label was drawn by the manual operator) and the false negative cases (FN, no lung detected by the network while the manual operator detected lung). From these numbers, the recall was calculated as TP/(TP + FN) and the precision as TP/(TP + FP).

Similarity of the segmentations from the manual operator and the network was assessed via the Sørensen-Dice similarity coefficient (DSC) [32] and the Hausdorff distance (HD) [33, 34]. For determining the latter, open source software was used ([35] software package downloaded from http://github.com/codalab/EvaluateSegmentation). In a DSC sub analysis, the 2D images were binned to cover fractions of 10% of the lung from the ventral to the dorsal parts of the chest in order to evaluate the performance of the CNN across the thorax. Results are expressed as means ± standard deviation. To check whether the network performs better in one of the two groups (healthy controls, CF-patients) values of DSC and HD were compared via a Mann–Whitney-U-test.

Obtained values for the lung volume and the fractional ventilation were compared via linear regression and a Bland–Altman analysis. Fractional ventilation was calculated as proposed earlier [4, 36], providing values in ml gas per ml lung tissue: Briefly, after a registration of all breathing states to one intermediate breathing state, signal intensity during inspiration was subtracted from the signal intensity in expiration and the resulting value was ultimately divided by the signal intensity in expiration.

Finally, the obtained lung volumes were also compared via a Wilcoxon-Singed-Rank-Test to find possible significant differences between the two approaches.

### Generalizability

In our center, CF has been the main focus of previous MR-UTE studies. Therefore, annotated images, i.e. images and corresponding manual segmentation masks, were available for this collective only. To test the network’s performance in patients suffering from other lung diseases, in particular in those with large consolidations, i.e. substantial changes in image contrast, possibly even interrupting the envelope of the lung, additional datasets were segmented both, manually and by the trained network: four datasets from patients with pneumonia and one dataset from a patient with a tumor in the lung. Such cases are typically challenging for algorithms based on region growing since the growing process would stop at the egde of consolidations with high signal intensities. In these datasets (488 images in total), only the DSC, HD and the lung volume were used for quality assessment since data has been obtained in only one breathing state and thus, calculation of fractional ventilation was not possible.

## Results

No significant differences in performance of the convolutional neural network were found between the datasets of the healthy controls and the CF-patients. Thus, results presented in the following paragraphs represent the entire test data set consisting of both, patients and controls. The average computation time needed for the segmentation of one 2D image was 87 ± 13 ms using the hardware described above.

### Performance of the CNN on general lung detection

In the test subset of 4920 images, a total of 3298 contained ground truth labels for lung tissue (67%). The model ended up with 3292 TP, 1614 TN, 8 FP and 6 FN cases. These numbers result in a recall of 99.8% and a precision of 99.8% in terms of general lung detection on 2D lung images from stack-of-spirals based UTE-MRI.

### Accuracy of lung detection by the CNN

The global accuracy was 99.9%. Accuracy for labels of the lung was 96.9% while for the background, the CNN reached a value of 100.0%.

Sørensen–Dice similarity coefficient for lung tissue and all 4920 coronal 2D images was 0.967 ± 0.076 with a 95% interval of confidence ranging from 0.965 to 0.970.

**Fig. 2:**
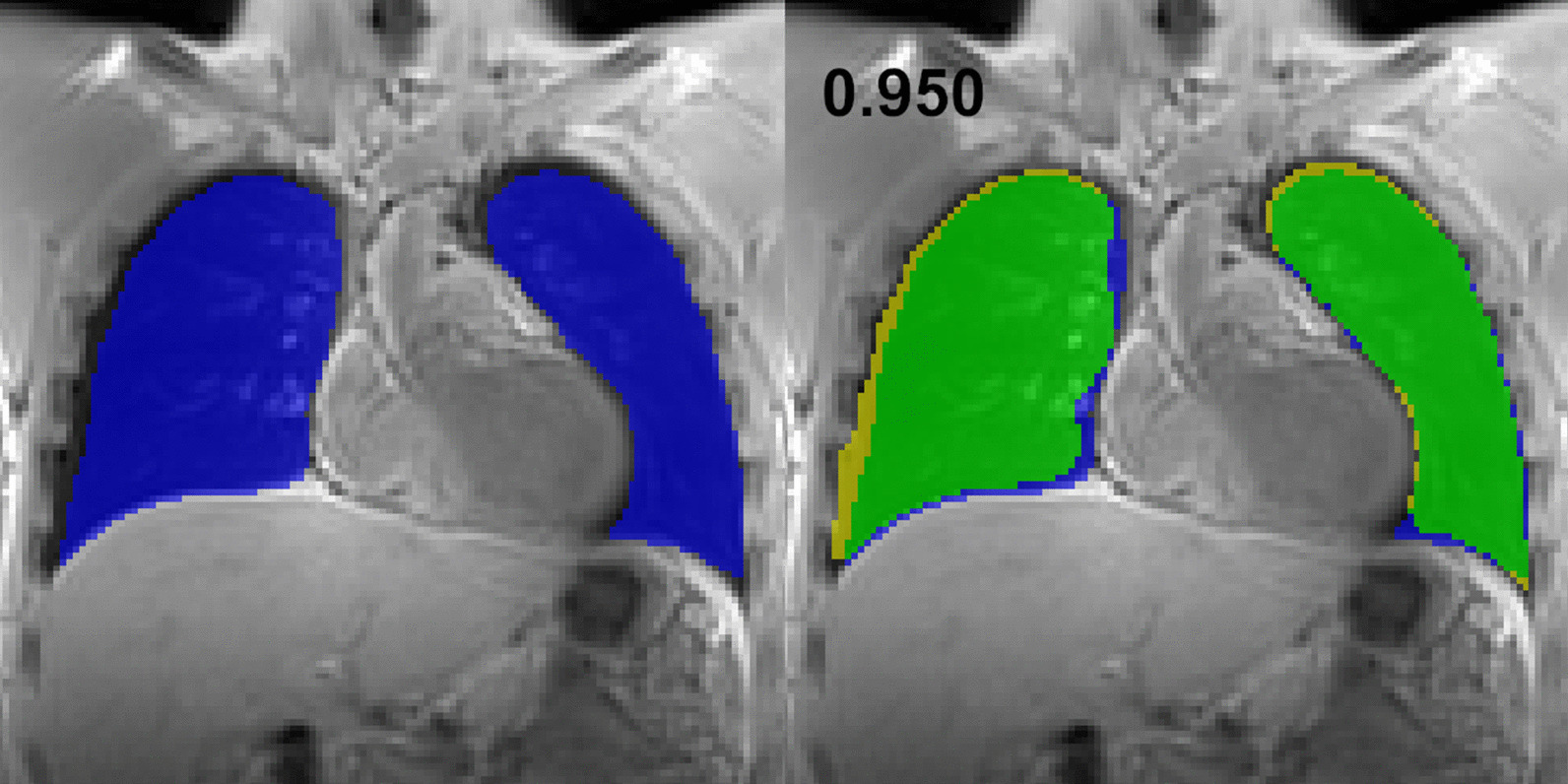
Morphologic image with superimposed labels obtained by applying the proposed model (left, same slice as in Fig. 1) and direct comparison of the two labels (right): yellow—manual, blue—automatic, green—consensus (DSC: 0.950)

**Fig. 3:**
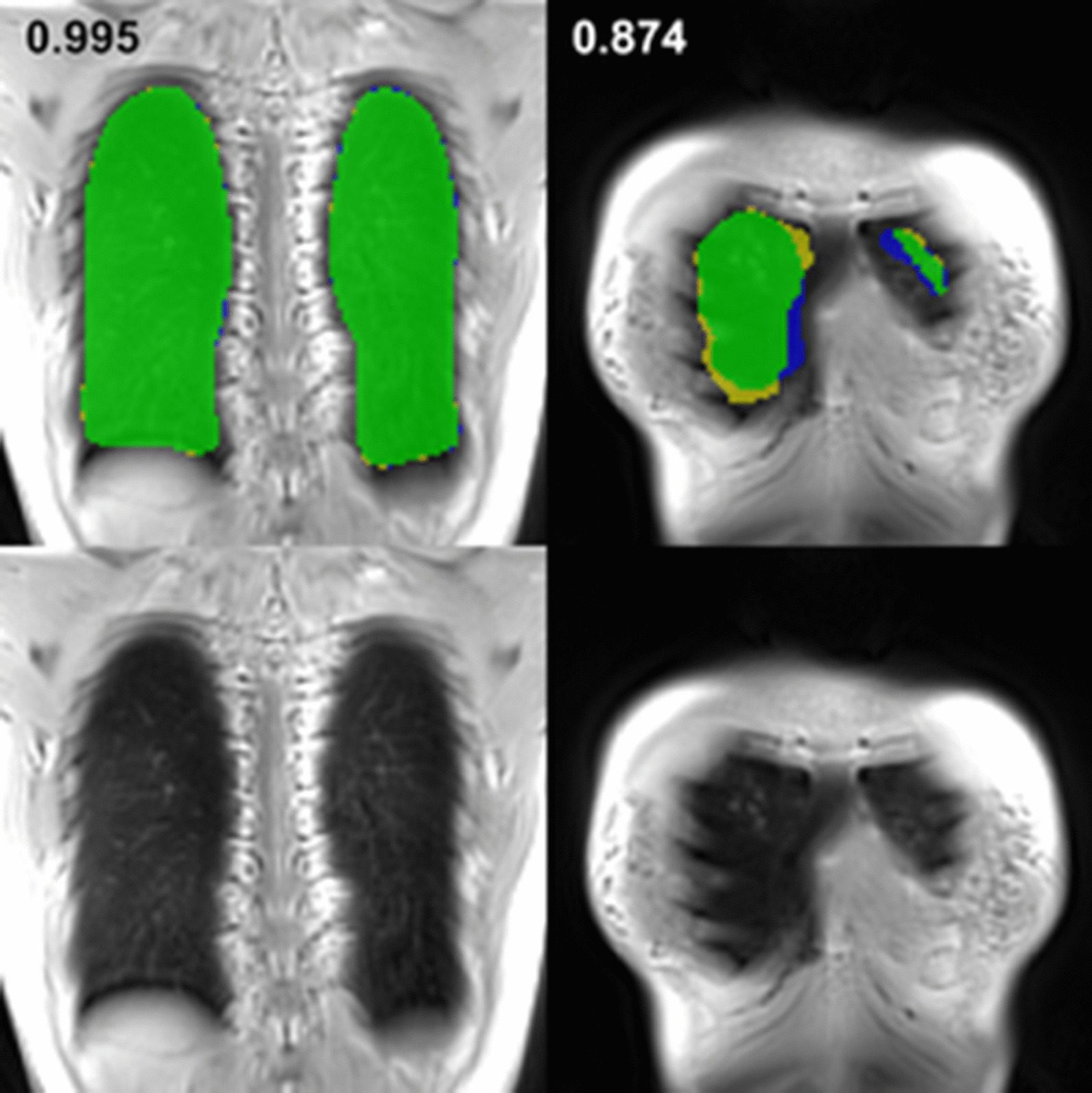
Examples of different segmentation results are presented for direct comparison (upper row, yellow—manual, blue—automatic, green—consensus). Corresponding anatomic images are presented in the lower row. Left: An almost perfect overlap of the manual and the automatic segmentation (DSC: 0.995). Right: A slice near the chest wall with low overlap between manual and deep learning based segmentation (DSC: 0.874)

Figure 2 (left panel) exemplarily shows a coronal slice of a 3D UTE MR dataset with the CNN-based segmentation superimposed and a direct comparison of the two techniques (right panel, DSC = 0.950). Values for the DSC of the different breathing depths are summarized in Table 1. In Fig. 3, representative examples of manual segmentations and according results obtained from model application are depicted for comparatively high (DSC: 0.995) and low similarity (DSC: 0.874).

**Table 1:** Presented are mean and corresponding standard deviation of the overall dice similarity coefficient and separated for the different breathing states

	Overall	Inspiration		Intermediate	Expiration	
		Deep	Normal		Normal	Deep
DSC						
Mean	0.967	0.975	0.974	0.977	0.977	0.978
std	0.076	0.043	0.048	0.045	0.042	0.036
HD (mm)						
Mean	4.1	4.0	4.4	4.1	4.0	4.4
std	4.4	0.6	1.3	0.6	0.6	0.9

Table 2 summarizes the mean DSC values for the different sections of the lungs in bins from the anterior to the posterior part. The similarity of the segmentations was notably lower in ventral slices of the lung. Except for the most ventral section (DSC: 0.957 ± 0.081) all sections showed DSC values over 0.960 with a range from 0.960 to 0.976.

**Table 2:** Division of the lung segmentation in parts of 10% starting at the ventral (1) and ending at the most dorsal (10) section. The most ventral part delivered a notably smaller mean DSC value with an also larger standard deviation. HD shows a similar behavior with higher values in the two most ventral parts

	1	2	3	4	5	6	7	8	9	10
DSC										
Mean	0.957	0.964	0.960	0.960	0.970	0.971	0.972	0.976	0.974	0.965
std	0.081	0.060	0.077	0.090	0.056	0.058	0.053	0.029	0.055	0.077
HD (mm)										
Mean	5.3	4.6	3.8	3.9	3.8	4.2	5.1	4.3	3.4	3.5
std	5.1	3.4	1.0	1.2	0.9	1.7	2.1	1.5	0.9	0.9

Hausdorff distance is only defined if both datasets contain the label for the class lung. Therefore, the 8 false positive and the 6 false negative slices were excluded from the calculation of the HD. The obtained values for HD are summarized in Table 2. On average, HD was 4.1 ± 4.4 mm. Like the DSC, HD was larger in the most ventral parts (up to 5.3 ± 5.1 mm) and between 3.4 mm and 4.3 mm in the sections containing primarily lung. Interestingly, in the central section—containing the heart in most of the cases—a HD of 5.1 ± 2.1 mm was calculated while DSC values did not drop significantly here.

### Lung volume and ventilation values

**Fig. 4:**
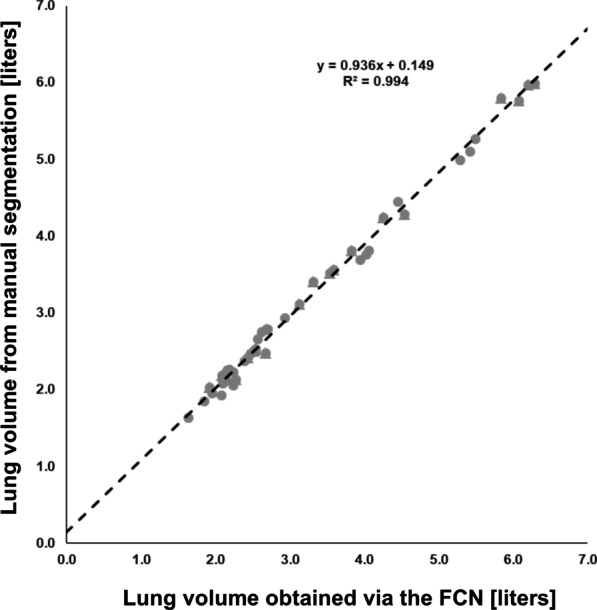
Scatterplot of the lung volumes obtained via the convolutional neural network vs. the lung volumes obtained via manual segmentation. Circles denote data from healthy volunteers while the triangles represent data from the patients suffering from cystic fibrosis. Linear regression was performed over all datapoints and resulted in a strong correlation

**Fig. 5:**
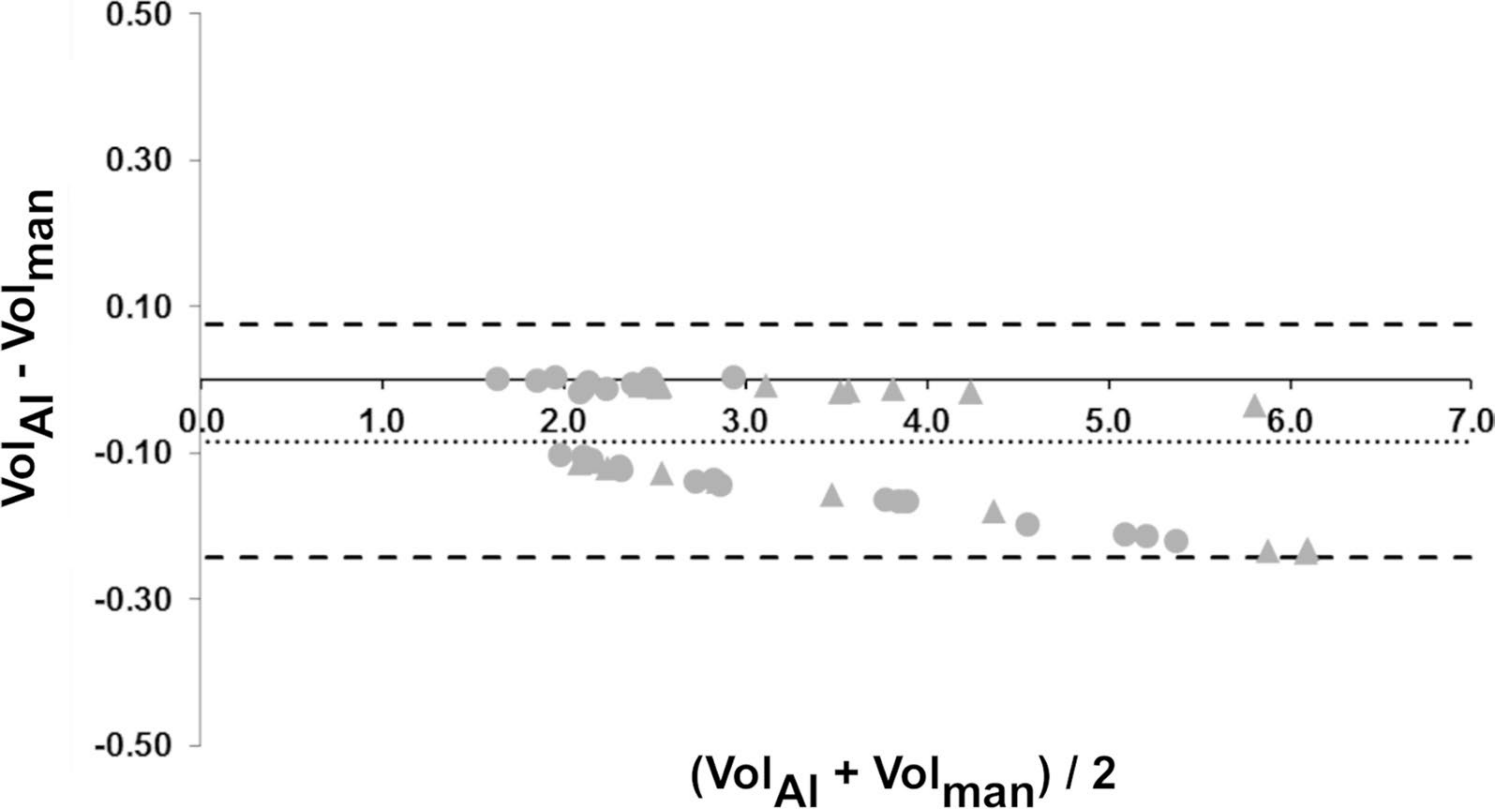
Bland–Altman-Plot of the comparison between manually and automatically obtained lung volumes. The dotted line represents the mean difference (-0.084 L) while the dashed lines define the lower (− 0.243 L) and upper (0.076 L) limit of agreement. Triangles represent data from CF-patients and circles denote data from the healthy volunteers

By use of the yielded segmentation, the total lung volume of each breathing depth and each volunteer of the test dataset was calculated. Results obtained when applying the CNN were compared to those based on manual post-processing. Linear regression yielded strong correlation (R^2^ = 0.994, Vol_CNN_ = 0.936 * Vol_man_ + 0.149) and the Bland–Altman analysis revealed a mean difference between manually and automatically obtained lung volumes of -0.084 L and limits of agreement of − 0.243 L and 0.076 L. Figure 4 shows a scatterplot and the linear regression line including the resulting R^2^ and equation while Fig. 5 presents the Bland–Altman plot for the comparison of the obtained lung volumes. The plot in Fig. 5 indicates a trend towards larger volumes for the manual segmentation. However, no significant difference was observed (p = 0.60).

**Fig. 6:**
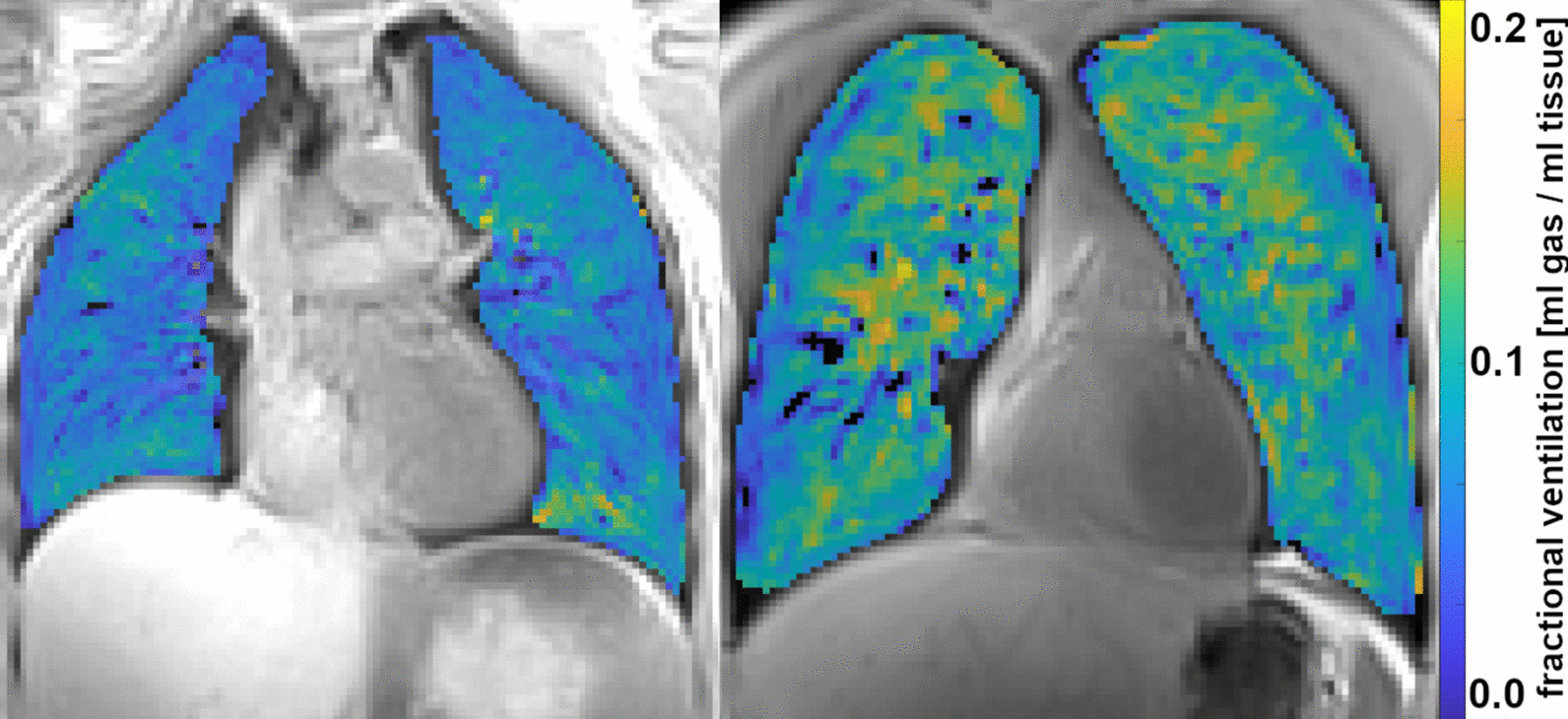
Exemplary ventilation maps of a healthy volunteer (left) and a patient with cystic fibrosis (right). The homogeneous appearance throughout the healthy lung is in great contrast to the expected heterogenous ventilation pattern of the CF-patient

Mean ventilation values calculated by means of the labels from manual segmentation was 0.12 ± 0.12 ml gas/ml tissue while CNN-based segmentation delivered values of 0.12 ± 0.08 ml gas/ml tissue, yielding a strong correlation (R^2^ = 0.993, Vent_CNN_ = 1.003 * Vent_man_ – 0.001). The Bland–Altman analysis resulted in a mean difference between the two techniques of 0.00 ml gas / ml tissue and limits of agreement of − 0.01 and 0.01 ml gas/ml tissue. Figure 6 exemplarily presents two fractional ventilation maps: a healthy volunteer (left) shows homogenous ventilation while the CF-patient (right) presents a more heterogeneous ventilation pattern, which is an expected behavior according to [20].

### Generalizability: datasets with consolidations inside the lung

In this additional set, application of the network was analyzed in a total of 488 images. A lung label was present in both manual and CNN-based segmentations of 343 images. In 11 images, the network detected lung tissue while no label was defined by the manual observer. Conversely, the network did not detect lung tissue in three images where the manual observer set a lung label. In 131 images no lung was segmented in both cases.

The mean DSC of the 345 images with lung labels in both segmentations was 0.930 ± 0.083 and the HD yielded 12.9 ± 16.2 mm. The mean difference in lung volume was 0.032 ± 0.048 L.

**Fig. 7:**
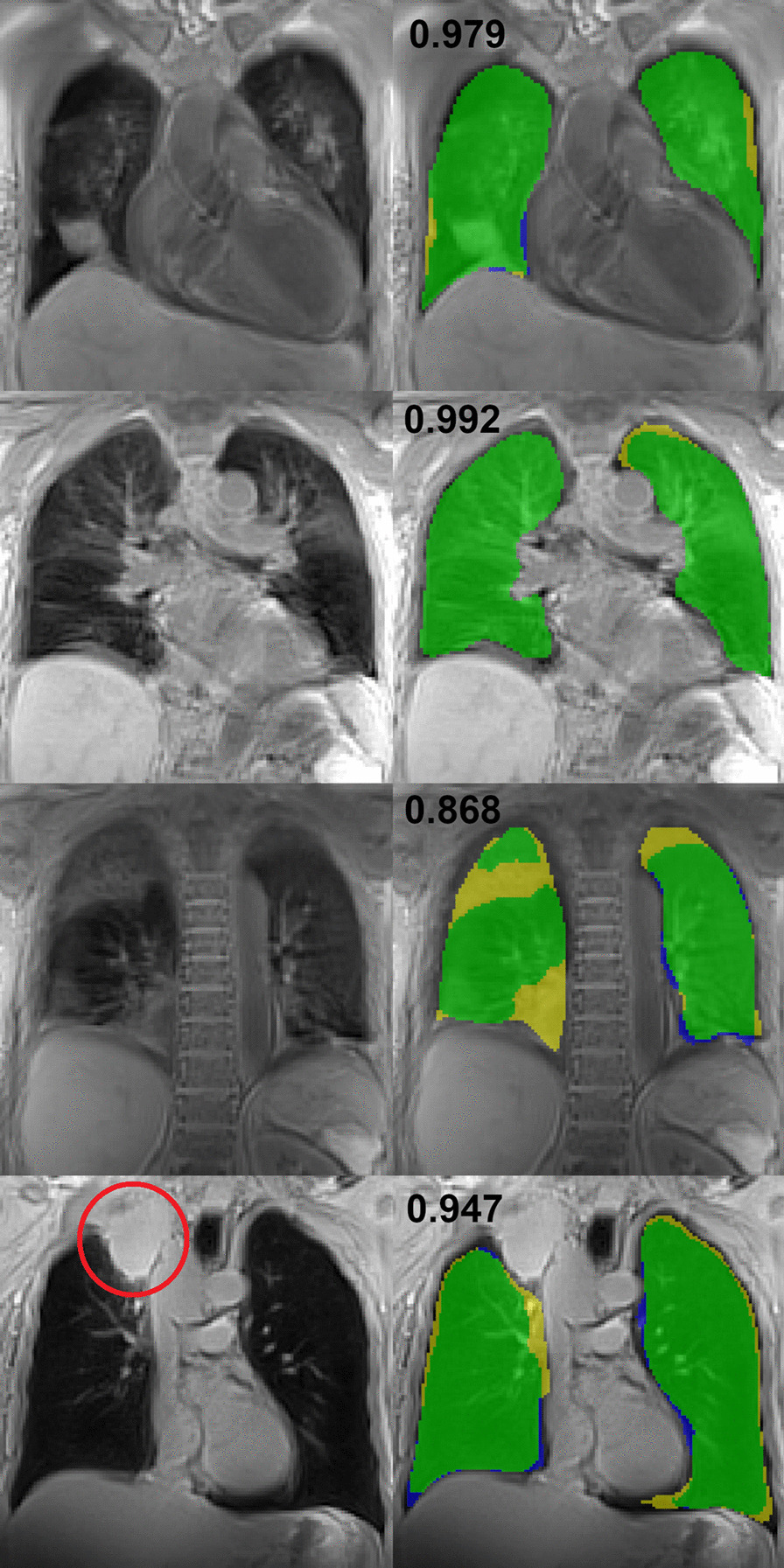
Results of the substudy investigating generalizability are presented. The left column shows the anatomical image while on the right the lung labels of both segmentations can be depicted (yellow—manual, blue—automatic, green—consensus). Numbers are the DSC for the respective slice. First row: image of a patient with pneumonia after stem cell transplantation.The consolidations on both lungs are segmented correctly by the network. Second row: example of another patient with pneumonia. Consolidations are segmented correctly. Third row: different slice of the same patient as in the row above. Due to very high signal intensity in the consolidations, the network failed to segment the lung correctly in this slice. Fourth row: patient with large tumor in the lung (indicated by the red circle on the anatomical image). The network did not include the tumor in the lung label. In this case, this is the intended behavior

The first row in Fig. 7 shows an example of a patient with consolidations as a consequence of pneumonia after stem cell transplantation. In this case, both segmentations show high similarity according to a DSC of 0.979.

Another example of a different patient is presented in the second row of Fig. 7 where the consolidations are correctly labeled as lung tissue (DSC: 0.992). A slice of the same patient was poorly segmented due to consolidations with high signal intensity (third row; DSC: 0.868). The last row of Fig. 7 shows an example of a patient with a tumor disrupting the lung envelope (red circle in the anatomical image). The manual observer as well as the neural network did not include the solid tumor in the lung label resulting in a DSC of 0.947 for this particular image.

## Discussion

The trained CNN enabled fully automatic and accurate segmentation in lung images obtained from stack-of-spirals-based UTE acquisitions. The Sørensen-Dice similarity coefficient, the Hausdorff distance as well as the strong correlation between manually and automatically derived lung volumes suggest an overall very good performance of the new approach with no significant drawbacks with respect to the cumbersome manual processing applied so far.

Slightly lower DSC values (0.957 ± 0.081) and higher HD values (5.3 mm ± 5.1 mm) were computed for the ventral part of the lung, however, without a large impact on calculation of the entire lung volumes, as reflected by a low mean difference between the two techniques in the Bland–Altman analysis (Fig. 5). The weaker performance in segmenting the ventral (see Fig. 3, right column) parts of the lung might be explained by different reasons: Differentiation of pulmonary parenchyma and thoracic wall is challenging for the human operator, especially because of partial volume effects and susceptibility artifacts at the tissue interfaces, which may lead to inconsistencies in the training data provided by a single manual operator. Secondly, those images of the periphery of the lungs are underrepresented in the training data, as each dataset comprises a high number of central slices and only a few slices at the edges, which additionally show a higher heterogeneity in their overall appearance.

In literature, artificial neural networks with a 3D architecture have been implemented and applied recently e.g. for the tracking of potential pulmonary perfusion biomarkers in chronic obstructive pulmonary disease patients [22] and for fully automated lung lobe segmentation in volumetric chest computed tomography images [24]. Both studies report a good overall performance of the networks (overall DSC 0.934 [22] and 0.948 [24]) but did not evaluate the performance with respect to possible dorsal or ventral inaccuracies leaving this comparison for further studies. In [37], 3D lung images were processed by a CNN trained with a template-based data augmentation strategy resulting in an overall very good DSC of 0.94 ± 0.02.

A previous study specifically focused on reducing the Hausdorff distance by means of a tailored loss function within the training process of a convolutional neural network [34]. The method was applied for investigations of the prostate (2D ultrasound and 3D MRI), the liver (3D computed tomography) and the pancreas (3D computed tomography). HD distances from 2.6 to 4.3 mm are reported which correspond to a comparable performance as observed for the method presented here.

In general, performance of a specific network always depends on the training data available and generalizability is not granted per se. Restricting parameters are signal-to-noise ratio, resolution, number of dimensions (2D vs. 3D) among several others. However, additional training of an existing model with own data (transfer learning) might allow the integration of previously published networks into one’s own clinical workflow or research environment.Our trained model can be downloaded here: https://github.com/expRad/LungSegmentation.

## Limitations

Even though the presented approach resulted in satisfying performance for the aimed purpose, with quality metrics within the range of the 3D approaches discussed above, a 3D CNN architecture might also be advantageous for the application focused in this study. With the size of the database acquired so far, however, sample size (~ 215 3D images) was estimated to be better suited for 2D processing, with a lower tendency towards overfitting. We therefore preferred splitting the reconstructed 3D images into 2D coronal slices, each representing a separate dataset for the 2D architecture used in our study. Nevertheless, the acquisition and inclusion of new cases is ongoing, such that the evaluation of a 3D CNN as a potentially better alternative represents an interesting study for future work. One additional potential issue of a 3D architecture remains the fact that the processing on a GPU requires a larger amount of memory, which is not always available.

In addition, a variety of alternative 2D architectures have been presented for semantic segmentation (Unet [38] or other fully convolutional networks [39]). However, a direct comparison of several currently popular architectures was beyond the scope of our study.

As can be seen in Figs. 1,2 and 3, we did not implement a separate step to eliminate vessels from the lung labels as performed in [27]. That might impair results for volume and ventilation even if the segmentations show high similarity to the manual post processing. However, previous studies showed that fractional ventilation can be reasonably calculated without a separate vessel extraction [4, 20, 28].

As already discussed, one crucial point during the development of such networks is the need for a sufficient amount of data for training, validation and testing. In the present study, data from 33 healthy volunteers and 20 CF-patients was available, which were exclusively scanned on the same 3 T scanner with the 3D stack-of-spirals UTE sequence protocol. While this led to satisfying results for the application targeted here, it might limit the generalization to acquisitions performed on scanners from different vendors, alternative UTE approaches and patient cohorts. The latter issue was assessed for our model by means of an additional substudy on subjects with pneumonia and a tumor in the lung. Overall DSC (0.930) was lower compared to the main study, however still within the range of earlier publications on automatic semantic segmentation of MR lung images. In detail, focal consolidations of medium size at the edges of the lung were interpreted in accordance between manual operator and CNN. Rather severe diffuse infiltrations covering large parts of the whole lung with strong changes in contrast led to incorrect segmentations by the neural network in scattered slices. Taking into account that the training data contained no relevant pathologies, these findings for extreme cases are not surprising. Nevertheless, the developed model can be subjected to a corresponding transfer learning with additional data to extend its applicability in this direction any time.

## Conclusions

In conclusion, the investigated convolutional neural network proved its capability for highly accurate segmentation of lung tissue in time-efficient 3D UTE acquisitions based on the stack-of-spirals k-space trajectory. The incorporation of the developed and evaluated method into the post-processing chain of the described MR-based functional lung imaging technique reduces manual interactions to a minimum and consequently facilitates the execution of large-scale studies in this field.

## Data Availability

The datasets used and/or analysed during the current study are available from the corresponding author on reasonable request. The trained segmentation model can be downloaded here: https://github.com/expRad/LungSegmentation.

## Abbreviations

COPD: Chronic obstructive pulmonary disease
CT: Computed tomography
MR: Magnetic resonance
SENCEFUL: Self-gated Non-Contrast-Enhanced Functional Lung imaging
UTE: Ultra-short echo time
ANN: Artificial neural networks
CNN: Convolutional neural network
CF: Cystic fibrosis
DSC: Sørensen-Dice similarity coefficient
ADAM: Adaptive moment estimation
HD: Hausdorff distance
Vol_CNN_: Lung volume obtained from automatically segmented images
Vol_man_: Lung volume obtained from manually segmented images
Vent_CNN_: Ventilation values obtained from automatically segmented images
Vent_man_: Ventilation values obtained from manually segmented images
